# Transcriptomic Analysis of *Pichia pastoris* (*Komagataella phaffii*) GS115 During Heterologous Protein Production Using a High-Cell-Density Fed-Batch Cultivation Strategy

**DOI:** 10.3389/fmicb.2020.00463

**Published:** 2020-03-20

**Authors:** Chengbo Zhang, Yu Ma, Huabiao Miao, Xianghua Tang, Bo Xu, Qian Wu, Yuelin Mu, Zunxi Huang

**Affiliations:** ^1^Engineering Research Center of Sustainable Development and Utilization of Biomass Energy, Ministry of Education, Yunnan Normal University, Kunming, China; ^2^School of Life Sciences, Yunnan Normal University, Kunming, China; ^3^Key Laboratory of Yunnan for Biomass Energy and Biotechnology of Environment, Yunnan Normal University, Kunming, China; ^4^Key Laboratory of Enzyme Engineering, Yunnan Normal University, Kunming, China

**Keywords:** *Pichia pastoris* (*Komagataella phaffii*), high-cell-density fed-batch cultivation, RNA-seq, MAPK signaling pathway, methanol induction, heterologous protein production

## Abstract

*Pichia pastoris* (*Komagataella phaffii*) is a methylotrophic yeast that is widely used in industry as a host system for heterologous protein expression. Heterologous gene expression is typically facilitated by strongly inducible promoters derived from methanol utilization genes or constitutive glycolytic promoters. However, protein production is usually accomplished by a fed-batch induction process, which is known to negatively affect cell physiology, resulting in limited protein yields and quality. To assess how yields of exogenous proteins can be increased and to further understand the physiological response of *P. pastoris* to the carbon conversion of glycerol and methanol, as well as the continuous induction of methanol, we analyzed recombinant protein production in a 10,000-L fed-batch culture. Furthermore, we investigated gene expression during the yeast cell culture phase, glycerol feed phase, glycerol-methanol mixture feed (GM) phase, and at different time points following methanol induction using RNA-Seq. We report that the addition of the GM phase may help to alleviate the adverse effects of methanol addition (alone) on *P. pastoris* cells. Secondly, enhanced upregulation of the mitogen-activated protein kinase (MAPK) signaling pathway was observed in *P. pastoris* following methanol induction. The MAPK signaling pathway may be related to *P. pastoris* cell growth and may regulate the alcohol oxidase1 (*AOX1*) promoter via regulatory factors activated by methanol-mediated stimulation. Thirdly, the unfolded protein response (UPR) and ER-associated degradation (ERAD) pathways were not significantly upregulated during the methanol induction period. These results imply that the presence of unfolded or misfolded phytase protein did not represent a serious problem in our study. Finally, the upregulation of the autophagy pathway during the methanol induction phase may be related to the degradation of damaged peroxisomes but not to the production of phytase. This work describes the metabolic characteristics of *P. pastoris* during heterologous protein production under high-cell-density fed-batch cultivation. We believe that the results of this study will aid further in-depth studies of *P. pastoris* heterologous protein expression, regulation, and secretory mechanisms.

## Introduction

*Pichia pastoris* (*Komagataella phaffii*) is a methylotrophic yeast species that has been shown to efficiently produce exogenous proteins. This species exhibits many of the advantages normally associated with *Escherichia coli* expression systems (Morton and Potter, 2000) while overcoming many of the deficiencies associated with *Saccharomyces cerevisiae* systems (Tran et al., 2017). *P. pastoris* has a strong alcohol oxidase1 (*AOX1*) promoter and performs post-translational folding and eukaryotic posttranslational modifications, such as glycosylation, disulfide bond formation, proteolytic modification, and targeted subcellular compartments. *P. pastoris* is suitable for the expression of high levels of foreign genes and is currently used to produce various industrial enzymes and pharmaceutical proteins (Hosseini et al., 2018; Wang Y. C. et al., 2019).

However, there are many limitations associated with industrial enzyme production using *P. pastoris* including the requirement for the flammable chemical methanol to induce expression, extended fermentation times, and low expression levels. Thus, it is imperative that further efforts are made to improve levels of expression exhibited by the analyzed strain. With this in mind, many researchers have conducted experiments that have resulted in the improvement of protein stability through rational design (Han et al., 2017, 2018), the development of strong promoters (Portela et al., 2018; Prielhofer et al., 2018; Ergun et al., 2019; Wang J. et al., 2019), the optimization of protein secretion (Barrero et al., 2018; Nguyen et al., 2018), the alteration of protein glycation in *P. pastoris* (Pekarsky et al., 2018), and co-expression of transcription factors (Chang et al., 2018; Dey et al., 2018). Furthermore, increasing productivity during scale-up of processes largely depends on trial and error; process optimization has been somewhat refined through the deployment of multifactorial experimental design (Ben Azoun and Kallel, 2017).

In recent years, several comprehensive, system-level approaches have been adopted to better understand the role of cellular networks for recombinant protein production. In a study performed by Liang et al. (2012), a comparative analysis between glycerol-mediated chemostat cultivation and methanol-mediated chemostat cultivation revealed that transcriptional regulation and ribosomal synthesis were the main biological processes that were stimulated when methanol was the sole carbon source. These results suggest that *P. pastoris* may exhibit reduced transcription for some less important native genes, thereby facilitating the increased generation of energy and substrates for the expression of the heterologous gene (Liang et al., 2012).

A fermentation and proteomic study by Vanz et al. (2012) reported that the degradation of recombinant protein may be initiated during the glycerol-fed phase. Specifically, their report suggested the modest increase in the methanol assimilatory enzyme level, as compared to the strong increase observed for methanol dissimilatory enzymes, likely results in methanol incorporation into the resultant product through metabolic enhancement of the methanol assimilatory pathway (Vanz et al., 2012).

Hesketh et al. (2013) investigated the physiological response of *P. pastoris* to heterologous expression using sorbitol and sorbitol plus methanol cultures; they observed that a marked, but temporary, stress response involving both UPR and ER-associated degradation pathways was observed during the transient phase between steady states for the different carbon sources. These results show that optimal production of heterologous protein could best be achieved by a continuous process, which minimizes the number of methanol-induced transient phases experienced by the cultures (Hesketh et al., 2013).

A study performed by Prielhofer et al. using *P. pastoris* cultures supplemented with different carbon substrates showed that although the cell growth rates were low, global translation was most prevalent in methanol-grown *P. pastoris* cells, followed by cells grown in excesses of glycerol or glucose. Although extensive transcriptional regulation was observed for cells grown on different carbon sources, transcript-specific translational responses were minimal. Moreover, a high proportion of mRNAs associated with polysomes in methanol-grown cells was also observed during the afore-mentioned study; this result indicates that high productivity during methanol induction is directly related not only to promoter strength but also to the growth conditions (Prielhofer et al., 2015). However, Cámara et al. (2017) found that the increased dose of *AOX1* promoter-regulated expression cassettes causes transcriptional attenuation of peroxisome biogenesis and methanol metabolism in *P. pastoris*; these phenomena occurred concomitantly with reduced secretion of the heterologous product. Therefore, because of the advantages associated with fed-batch cultures (Pedersen et al., 2000), high-cell-density fed-batch cultivation encompassing four stages, namely, yeast cell culture phase, glycerol feed, GM phase (Cos et al., 2006; Potvin et al., 2012), and methanol induction phases is desirable for analyzing the synthesis of recombinant proteins and investigating responses to various carbon sources.

In this study, we performed fermentation reactions using a previously constructed high-yield phytase-producing genetically engineered strain using high-cell-density fed-batch cultivation. Unlike previous studies, we utilized a GM stage prior to the methanol growth stage. In addition, we investigated gene expression in this strain at each fermentation stage using RNA-Seq. We found that the addition of the GM stage in our fed-batch culture may help to alleviate the adverse effects of methanol on *P. pastoris* cells, and this may also be conducive to the production and secretion of recombinant proteins. Furthermore, many metabolic pathways, including carbon metabolism, oxidative stress response, MAPK signaling pathway, UPR and ERAD pathways, and the autophagy pathway, were explored by transcriptomic data analysis. This study provides us with important information that will facilitate further in-depth studies pertaining to *P. pastoris* foreign protein expression, regulation and secretory mechanisms.

## Materials and Methods

### Strain and Fermentation

The Mut^+^ strain of *Pichia pastoris* (*Komagataella phaffii*) GS115 (His4) was used to express *Aspergillus niger* PhyA phytase under the control of the inducible AOX1 promoter. The alpha-mating factor secretion signal of *S. cerevisiae* was also incorporated into the plasmid construct (Han et al., 2018). *P. pastoris* was grown in liquid and solid media during fermentation in accordance with the procedure used by Rahimi et al. (2019) with slight modifications.

The resultant recombinant *P. pastoris* was inoculated in a 1-L Erlenmeyer flask with 200 mL of standard medium (1.34% [w/v] yeast nitrogen base, 2% [w/v] peptone, 1% [w/v] yeast extract, 0.4 mg/L biotin, and 100 mM potassium phosphate [pH 6.0]) containing 1% (w/v) glycerol (Buffered Glycerol-complex Medium). The inocula were placed in an incubator shaker at 29°C/250 rpm for 36–38 h until a wet cell weight of 30 g/L was achieved.

Seed-scale batches were fermented in two stages, namely, using 50-L and 500-L fermenters. The seed-scale batch medium used (50 L) contained the same components as the standard medium in the 1-L Erlenmeyer flask. The prepared pre-culture containing 30 L of sterilized fermentation media was used to inoculate a 50-L fermenter. Fermentation was conducted in a bioreactor in batch mode at 30°C/350 rpm and a pH of approximately 4.5 with the addition of 20% w/v ammonia solution until wet cell weight reached 61 g/L. The seed-scale batch (500 L) contained 300 L of sterilized fermentation media. Each liter of fermentation culture media contained 5.0 mL of H_2_SO_4_, 40 mL of glycerol, basal salt medium (comprising 4.13 g of KOH, 14.9 g of MgSO_4_⋅7H_2_O, 18.2 g of K_2_SO_4_, 0.93 g of CaSO_4_, 26.7 mL of 85% H_3_PO_4_) (Stratton et al., 1998), trace mineral mix (65.0 g of FeSO_4_⋅7H_2_O, 20.0 g of ZnCl_2_, 0.5 g of CoCl_2_, 0.02 g of H_3_BO_3_, 0.2 g of Na_2_MoO_4_⋅2H_2_O, 3.0 g of MnSO_4_⋅H_2_O, 0.08 g of NaI, 6.0 g of CuSO_4_⋅5H_2_O), and glucose-vitamin mix (128 mg of C_6_H_12_O_6_, 12.8 mg of C_18_H_32_CaN_2_O_10_, 64 mg of K_2_HPO_4_, 12.8 mg of C_8_H_9_NO_3_⋅HCl, 12.8 mg of D-biotin, 0.2 g of nicotinic acid [vitamin B3], and 3.2 mg of thiamine hydrochloride [vitamin B1]). Fermentation in the bioreactor was conducted in batch mode at 30°C/250 rpm and a pH of approximately 4.5 with the addition of 20% w/v ammonia solution until wet cell weight reached 64 g/L.

The fermenter (10,000 L) for the yeast cell culture (10,000 L) contained 5000 L of sterilized fermentation media. The medium used was the same as that used for the 500-L seed-scale batch. Fermentation in the bioreactor was conducted in fed-batch mode with the addition of 20% w/v ammonia solution at 30°C and a pH of approximately 4.7–5. The rotational speed of the impeller was 160 rpm and the air flow was controlled at 180 cm^3^/h during fermentation. This fermentation process consisted of four steps; the first step, the glycerol batch cultivation step, was required to facilitate the growth of *P. pastoris* and was conducted over approximately 12 h until wet weight = 47.2 g/l. Next, the fed-batch cultivation phase, which encompassed the second to the fourth stages, was initiated. In the second stage, dissolved oxygen (DO) levels reached 100% saturation and were maintained at these levels for 2 h. We subsequently injected 250 L of 50% glycerol into the media at a flow rate of 50 L/h for 5 h until a wet weight of 140–150 g/l was reached; DO was maintained at 20–30% saturation during the entirety of the second step. In the third stage, we injected 250 L of 50% glycerol-20% methanol mixture into the glycerol-methanol feed at a flow rate of 50 L/h for 5 h until a wet weight of 140–160 g/l was achieved. In the fourth stage, the methanol feed was injected with 15 L/h methanol. Fed-batch fermentation was conducted with the addition of 20% v/v ammonia under approximately constant DO (20–30%) at 30°C and a pH of approximately 4.7–5. The fourth stage lasted for 120–150 h. The reaction was performed using the DO cascade mode with the agitation speed adjusted to 150–180 rpm and inlet air to 1–1.2 VVM. The physical pressure of the entire fed-batch culture stage was maintained at 0.05 MPa.

### RNA-Seq Sample Preparation

Samples from the yeast cell culture phase, second phase, third phase, and fourth phase at different time points were obtained and centrifuged, and the supernatant was removed. The cells were immediately frozen in a liquid nitrogen tank, and the samples were collected and sent to Novogene Technology Co., Ltd (Beijing, China) for RNA extraction and transcriptome sequencing.

### RNA Sequencing and Data Analysis

For Illumina sequencing, the extraction, quantification and identification of total RNA was performed in accordance with procedures outlined by Liang et al. (2012). The cDNA library was constructed using a method published by Zou et al. (2016). The cDNA was subsequently sequenced on an Illumina NovoSeq 6000 platform. Quality control of raw data (raw reads) was performed according to the standard methods of Novogene Technology Co., Ltd (Yu et al., 2014).

Reference genome and gene model annotation files were directly downloaded from a genome website^1^. A reference genome index was generated, and paired-end clean reads were aligned to the reference genome using Hisat2 v2.0.5. Hisat2 was selected as the mapping tool because it can generate a database of splice junctions based on gene model annotation files; thus, this tool achieves more optimal mapping results than other non-splice mapping tools. All of the genes of interest in this study were compared and revised based upon the annotations of the three curated reference genomes of CBS7435 (Supplementary Tables S1–S4; Valli et al., 2016). The gene expression data generated during this study have been submitted to the Gene Expression Omnibus (GEO) database of NCBI; a GEO accession number GSE142326 was assigned to these data^2^.

We performed differential gene expression analysis based on the research method of Liang et al. (2012). We subsequently used the clusterProfiler R package to evaluate the statistical enrichment of differentially expressed genes in KEGG pathways^3^.

### Enzyme Activity

Phytase activity was measured from the culture supernatant using a modified molybdenum blue method (Lahti and Heinonen, 1981). This process was conducted at 700 nm using colorimetrical quantification of free phosphorus released during phytate hydrolysis and ammonium molybdate as the color reagent. The standard enzymatic reaction was carried out at 40°C for 30 min in a reaction mixture containing the prepared enzyme (200 μl) and substrate solution (800 μl) with 4 mM CaCl_2_, 100 mM Tris-HCl buffer (pH 6), and 1 mM sodium phytate. The reaction was terminated by adding 1 mL of 5% (w/v) trichloroacetic acid. The concentration of released inorganic phosphate was determined by adding 1 mL colorant, which consisted of 3.5% (v/v) sulfuric acid, 0.54% (w/v) ferrous sulfate and 1.2% (w/v) ammonium molybdate. Measure the absorbance at 700nm.

## Results

### A Fed-Batch Culture System Used to Explore the Effects of Inducing Heterologous Expression of Phytase in *P. pastoris*

*Pichia pastoris* is mainly used for the industrial production of enzymes and pharmaceutical proteins. By comparing recent transcriptomics or proteomics studies pertaining to the expression of recombinant proteins in *P. pastoris* (Liang et al., 2012; Vanz et al., 2012; Hesketh et al., 2013), we identified studies where fermentation was conducted through direct methanol culture or the addition of methanol to glycerol-mediated growth cultures. However, it should be noted that the results of laboratory studies can be inconsistent with the results of large-scale fermentations. In addition, methanol addition can induce the expression of recombinant proteins, but can also have negative impacts including reductions in the growth rate of *P. pastoris*, the inability of recombinant proteins to fold efficiently, and the degradation of recombinant proteins (Liang et al., 2012). Therefore, we utilized 10,000 L of a high-cell-density fed-batch cultivation for phytase heterologous production; we added a GM phase (Cos et al., 2006; Potvin et al., 2012) between the glycerol growth stage and the pure methanol addition stage to improve the adaption of *P. pastoris* to the methanol environment. The latter addition was based upon previous data generated from a 100-L fermentation (Supplementary Figure S1) and data generated during previous studies (Jordà et al., 2013; Rußmayer et al., 2015).

**FIGURE 1:**
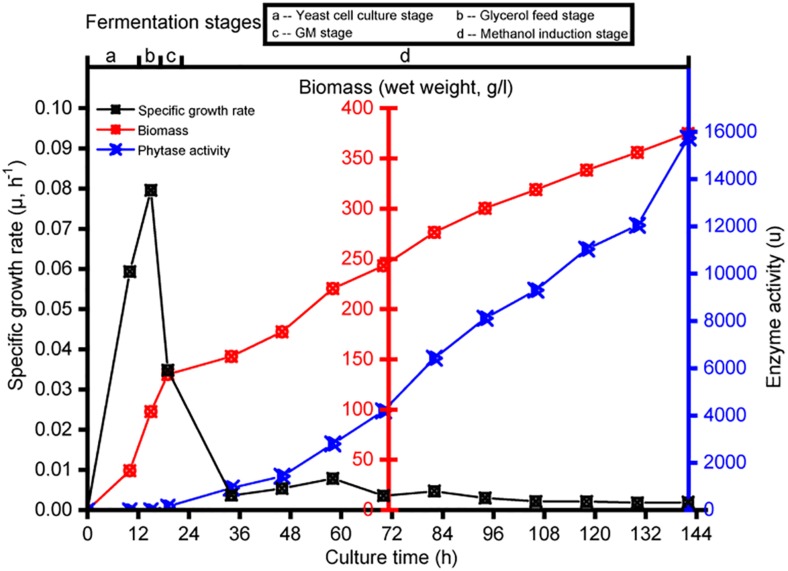
Changes in specific growth rate, biomass, and enzyme activity during the fermentation in *Pichia pastoris*. Detection sample points from left to right are B_10, G_3, GM_2, M_12, M_24, M_36, M_48, M_60, M_72, M_84, M_96, M_108, and M_120.

Transcriptomics analysis was used to investigate the physiological response of *P. pastoris* to the various carbon sources. Each culture was sampled at intervals; samples were taken from the yeast cell culture growth fermentation at 10 h (designated B_10), glycerol growth at 3 h (designated G_3), glycerol and methanol mixture addition culture at 2 h (designated GM_2), and pure methanol addition culture at 12-h intervals (designated M_12, M_24, M_36, M_48, M_60, M_72, M_84, M_96, M_108, and M_120). Strain-specific growth rate, biomass, and enzyme activity data for the different cultures are shown in Figure 1. No enzyme activity was observed during the yeast cell culture and glycerol feed phases. Compared with observations made during the initial methanol induction, the enzyme activity after methanol induction at ≥12 h reached 15,738 U (Figure 1).

### Analysis of Metabolic Pathway Responses of *P. pastoris*

**FIGURE 2:**
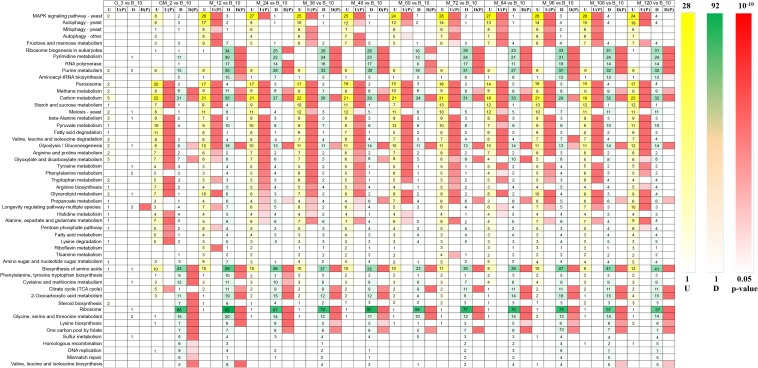
Heatmap of upregulated and down-regulated genes that were significantly enriched in 52 metabolic pathways of *Pichia pastoris*. U, upregulated gene number, U(p): *p*-Value of upregulated genes enriched in metabolic pathway, D, down-regulated gene number, D(p): *p*-Value of down-regulated genes enriched in metabolic pathway.

Phytase-producing *P. pastoris* was used for fermentations that utilized high-cell-density fed-batch cultures in a 10,000-L fermenter. We investigated the gene expression of this strain using RNA-Seq during the yeast cell culture phase, glycerol feed phase, glycerol-methanol feed phase, and at 12-h intervals during the methanol induction phase. RNA-Seq results indicated that significantly altered genes (*p*-Value < 0.05 and log2 fold changes > 0.50/log2 fold changes < −0.50) were enriched in 52 KEGG metabolic pathways (Figure 2). As shown in the heat map reporting the gene number changes for each KEGG metabolic pathway, no genes were significantly upregulated during the glycerol growth stage compared with yeast cell culture stage, the genes that were significantly upregulated from the GM phase were mainly involved in peroxisome, starch and sucrose metabolism, and fatty acid degradation (*p*-Value < 0.05 and log2 fold changes > 0.50). However, in contrast to a fermentation study performed without a GM stage by Li et al. (2019), in this current study, genes that were enriched in the MAPK signaling pathway were significantly upregulated in the methanol induction stage (*p*-Value < 0.05 and log2 fold changes > 0.50), and genes involved in autophagy in yeast, mitophagy in yeast, autophagy–other, and fructose and mannose metabolism were significantly upregulated almost throughout the entirety of the methanol induction phase (*p*-Value < 0.05 and log2 fold changes > 0.75). Among these processes, the upregulation of genes in the MAPK signaling pathway was the most significant (*p*-Value < 10^–5^ and log2 fold changes > 0.75) (Figure 2). A previous study reported that the MAPK signaling protein in *P. pastoris* may be involved in cell wall integrity (Cosano et al., 2001). Thus, understanding the MAPK signaling pathway in *P. pastoris* can help determine the physiological, biochemical and genetic regulatory responses of *P. pastoris* to high methanol concentrations.

Ribosome, amino acid biosynthesis, pyrimidine metabolism, purine metabolism, cysteine and methionine metabolism, 2-oxocarboxylic acid metabolism, and glycine, serine and threonine metabolism harbored genes that were significantly down-regulated during the GM phase (*p*-Value < 0.05 and log2 fold changes < −1.00). Genes involved in ribosome biogenesis in eukaryotes, RNA polymerase, and the citrate cycle (TCA cycle) were significantly down-regulated in the methanol induction phase (*p*-Value < 0.05 and log2 fold changes < −0.50). Among these pathways, the number of genes that were down-regulated in the ribosome pathway was the most significant (*p*-Value < 10^–14^ and log2 fold changes < −0.50); furthermore, ribosome biogenesis in eukaryotes was the most significantly down-regulated activity during the methanol induction stage (*p*-Value < 0.01 and log2 fold changes < −0.50) (Figure 2). These results suggest that *P. pastoris* may exhibit reduced transcription for some less important native genes, thereby facilitating the increased generation of energy and substrates for the expression of the heterologous gene (Liang et al., 2012).

Starting from the adaptation period following the addition of the GM phase, genes involved in methanol metabolism, carbon metabolism, and glycolysis/gluconeogenesis were both significantly upregulated and significantly downregulated (Jordà et al., 2013). These results indicate that pathways related to carbon metabolism are especially significant during heterologous expression activities (Rußmayer et al., 2015; Li et al., 2019).

Therefore, the intracellular transcriptome of *P. pastoris* showed significant changes in response to the transition from growth on glycerol to production and growth on methanol (Figure 2); these observations are similar to those made in a previous proteomic study of *P. pastoris* (Vanz et al., 2012). As noted above, differences in metabolic pathways between the GM growth phase and the methanol induction growth phase suggest that addition of the GM phase could facilitate the adaptation of *P. pastoris* growth during the methanol induction phase.

### Carbon Metabolism

The carbon metabolism pathways of *P. pastoris* predominantly include methanol metabolism, glycolysis, the TCA cycle, the pentose phosphate pathway, and ethanol metabolism. In particular, metabolic processes, such as methanol metabolism, glycolysis, the TCA cycle, and the pentose phosphate pathway, produce a large number of intermediate metabolites, providing substrates for other metabolic processes.

**FIGURE 3:**
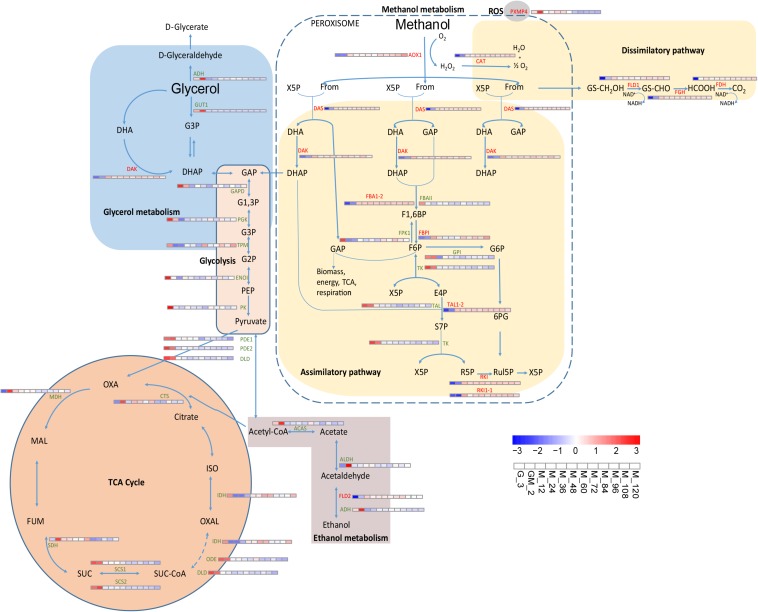
Changes in proteins involved in carbon metabolism and oxidative stress metabolic pathway. Rußmayer et al. (2015) was the first study to report that the methanol assimilation pathway localized to peroxisomes.

In the glycerol growth phase, the intracellular metabolic pathways are predominantly concentrated in oxidative phosphorylation, glycolysis, the TCA cycle and electronic respiratory chain, while during the methanol induction phase, intracellular metabolic pathways are mainly concentrated in the methanol metabolism pathway (Ren et al., 2003). After assimilation and absorption of glycerol and methanol, respectively, the intermediate metabolite undergoes carbon metabolism, which is predominantly mediated through the TCA cycle. To further analyze carbon metabolism, a schematic map of this process was generated based on studies that previously investigated the *P. pastoris* metabolic pathway (Van Der Klei et al., 2006; Vanz et al., 2012; Jordà et al., 2013; Rußmayer et al., 2015; Vogl et al., 2016) and the transcriptome data generated during this study (Figure 3).

The methanol absorbed by the cells was metabolized by the methanol pathway in the peroxisome. The H_2_O_2_ produced by the latter metabolism affected O_2_ formation following the activity of the ROS pathway (Van Der Klei et al., 2006). Methanol is oxidized to formaldehyde that goes onto form CO_2_ through dissimilation (Li et al., 2019); however, some of the latter formaldehyde enters the pentose phosphate pathway, and one molecule of methanol finally produces one-third of a molecule of glyceraldehyde 3-phosphate (GAP) for biomass synthesis (Figure 3).

Upon utilization of glycerol and methanol as the sole carbon sources, the *P. pastoris* cell growth status was altered, and the intracellular metabolic system was partially adjusted (Prielhofer et al., 2015). Conversely, when *P. pastoris* cultures use glycerol as the sole carbon source, the external carbon source is predominantly assimilated through glycerol metabolism (Figure 3). In the glycerol metabolism pathway, glycerol is irreversibly catalyzed by glycerol kinase (GUT1) to form glycerol 3-phosphate (G3P), which is subsequently transformed into dihydroxyacetone phosphate (DHAP) and glyceraldehyde 3-phosphate (GAP); the latter products then enter glycolysis to form pyruvate, thereby generating acetyl-CoA, which subsequently enters the TCA cycle. Glycerol promotes cell growth, and during this growth stage, foreign proteins are barely expressed (Orman et al., 2009). We compared the data generated during this study to those published by Prielhofer et al. (2015) and Rußmayer et al. (2015), where it was suggested that methanol utilization and peroxisome genes were inhibited by glycerol. However, in addition to glycerophosphate dehydrogenase (GPD) and triose phosphate isomerase (TPI1), the levels of glycerol metabolism-related proteins like GUT1 and alcohol dehydrogenase (NADP^+^) (ADH, *PAS_chr1-1_0357*) exhibit significant changes throughout the fed-batch culture (Figure 3). GUT1 (log2 fold changes < −3.70) and ADH (log2 fold changes < −0.60) were obviously upregulated during the adaptation period of glycerol and methanol mixture addition (GM), and then significantly down-regulated during the methanol induction period (from M_12 to M_120) (*p*-Value < 0.001) (Figure 3 and Supplementary Table S1). Similar changes as those observed for GUT1 were also observed for TCA cycle-related proteins like MDH, SDH, SCS1, SCS2, DLD, ODE, and CTS and the ethanol metabolism-related proteins like ACAS and ADH (Figure 3 and Supplementary Table S1). Moreover, proteins PDE1, PDE2, and DLD, which catalyze the conversion of pyruvate to acetyl-CoA, proteins GAPD, PGK and ENOI involved in glycolysis (Figure 3 and Supplementary Table S1). These results indicate that during the adaption period of GM, *P. pastoris* cell growth still occurs although a limited number of recombinant proteins are produced (Figure 1).

Alcohol oxidase1, which catalyzes the formation of formaldehyde from methanol, is significantly upregulated during the addition of the GM phase (*p*-Value < 0.001 and log2 fold changes ≈ 6.30) (Supplementary Table S1); this event is further enhanced during the methanol induction period. CAT, FLD1, FGH, and FDH involved in the methanol dissimilatory pathway, as well as DAS and DAK involved in the methanol assimilatory pathway, were all enhanced in a similar manner to AOX1 (Jordà et al., 2013; Rußmayer et al., 2015). These results suggest that the damage caused to *P. pastoris* cells by the high concentration of methanol is likely to become reduced during the methanol induction period after the low-concentration methanol adaptation period. Several proteins (DAS, DAK, FBPI, and RKI) in the methanol assimilation pathway are also moderately upregulated compared with the significant upregulation of proteins related to the methanol dissociation pathway (*p*-Value < 0.05 and log2 fold changes > 1.40) (Supplementary Table S1). In accordance with previous reports, the latter observations suggest that methanol is not only oxidized by *P. pastoris* during the dissimilation pathway to facilitate the generation of energy following re-oxidation of NADH in the respiratory chain but also for incorporation into biomass in the assimilation pathway (Vanz et al., 2012; Jordà et al., 2013; Rußmayer et al., 2015).

According to the study of Rußmayer et al., there are two categories of enzyme isoforms responsible for the assimilatory pathway. The first of these categories (Fba1-1, Tal1-1, Rki1-1, and Rpe1-1) targets the cytosol while the second (Fba1-2, Tal1-2, Rki1-2, and Rpe1-2) targets the peroxisome. Upon growth in methanol-supplemented media, the peroxisomal-targeting proteins were induced, and the assimilation of formaldehyde was localized to the peroxisome (Rußmayer et al., 2015). In this study, we found that there were two sets of these enzymes (Figure 3). We were able to differentiate between the isoforms using RNAseq. Fba1-2 and Tal1-2 were significantly upregulated throughout the methanol induction phase, while FBAII (Fba1-1) and TAL (Tal1-1) were significantly downregulated (Figure 3 and Supplementary Table S1). Although Rki1-1 and RKI (Rki1-2) were both upregulated in this study, compared with the upregulation of Rki1-1, RKI (Rki1-2) was significantly upregulated (Log2 fold changes > 5 and *p*-Value < 0.001). However, ribulose-5-phosphate 3 epimerase (Rpe1-1) and Rpe1-2 were not differentially regulated during the same phase. Nonetheless, compared to the unannotated genes, PAS_FragB_0022, PAS_chr2-1_0249 and PAS_chr1-1_0108 were up-regulated in glucose-limited fermentation studies (Prielhofer et al., 2015). Each of the latter proteins containing predicted PTS1 targeting signals (Neuberger et al., 2003) were down-regulated during the methanol induction phase of this study compared with the GM phase (Supplementary Table S1). In accordance with the study by Rußmayer et al., Fba1-2, Tal1-2, and Rki1-2 each contained a PTS1 peroxisome-targeting signal, suggesting that these proteins might be involved in separate peroxisome methanol assimilation pathways. Thus, our findings, where the entire methanol assimilation pathway is localized to peroxisomes, rather than using part of the cytosolic pentose phosphate pathway for xylulose-5-phosphate regeneration, are consistent with the study by Rußmayer et al.

### Oxidative Stress Response

**FIGURE 4:**
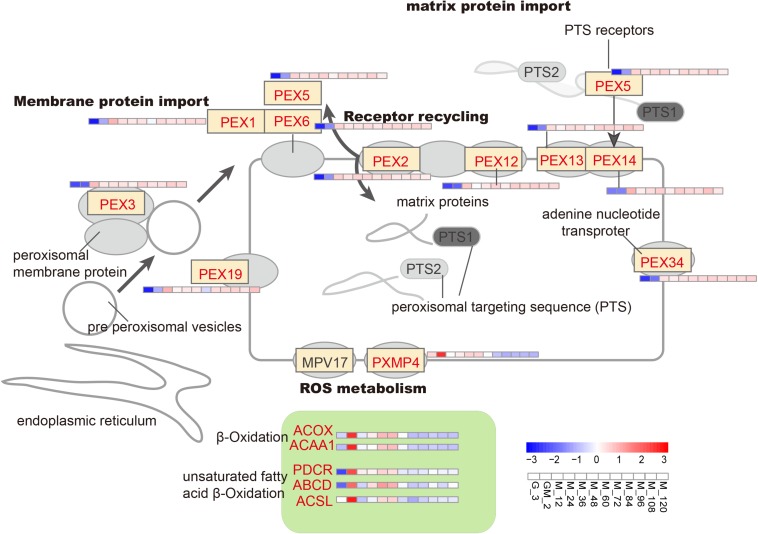
Changes in proteins involved in the oxidative stress metabolic pathway.

It has previously been demonstrated that *P. pastoris* requires greater native peroxidase levels to resist toxicity to methanol when methanol is added into chemostat cultures (Liang et al., 2012). From the GM phase, almost all genes controlling the development and function of peroxisomes are gradually upregulated compared with the glycerol growth stage (detection sample is G_3) (Figure 4 and Supplementary Table S1). However, it should be stated that the latter results differ from those published by Li et al. (2019). Other peroxidated molecules are also generated during the oxidation of methanol-reactive oxygen species (e.g., hydrogen peroxide); these latter molecules require removal to prevent or minimize cellular damage (Vanz et al., 2012). At least two peroxisomal enzymes including catalase (CAT), which removes hydrogen peroxide (Horiguchi et al., 2001a), and a glutathione peroxidase or peroxiredoxin (PMP20), which removes peroxidated molecules, e.g., lipid hydroperoxides are involved in the removal of reactive oxygen species (Horiguchi et al., 2001b; Vanz et al., 2012). However, in this study, compared with the significant increase of CAT (*p*-Value < 0.001 and log2 fold changes > 2.00) (Supplementary Table S1), PMP20 was not significantly upregulated throughout the fed-batch culture process. The presence of PMP20 is essential for the maintenance of peroxisomal membrane integrity during growth on methanol (Horiguchi et al., 2001b; Bener Aksam et al., 2008; Vanz et al., 2012).

Peroxisome proteins are encoded by nuclear genes, synthesized in the cytoplasm, and then introduced into peroxisomes. Two peroxisome-targeting signal genes (*PTS1* and *PTS2*) are critical for classifying proteins into this organelle. *PEX5* and *PEX7* encode receptors for *PTS1* and *PTS2*, respectively (Veenhuis et al., 2015). However, *PEX7* has been shown not to undergo change before or after the addition of methanol; this is consistent with the limited proportion peroxisomal matrix proteins, which contain the *PTS2* receptor (Liang et al., 2012). *PEX1*, *PEX3*, *PEX6*, *PEX13*, and *PEX14*, which are involved in peroxisomal membrane or matrix protein import, and *PEX2* and *PEX12*, which participate in receptor recycling, were upregulated (Figure 4), showing that there is a requirement for the introduction of matrix proteins into peroxisomes (Rußmayer et al., 2015); these results are consistent with Liang’s study (Liang et al., 2012).

In yeast, β-oxidation (the main pathway for fatty acid degradation) is performed exclusively in the peroxisomes. The acetyl-CoA that is generated as a result of this process can be redirected to the glyoxylate cycle or transported to the mitochondria via the carnitine shuttle, where it can be utilized a source of energy and an anaplerotic supply of building blocks (Cámara et al., 2017). Long-chain acyl-CoA synthetase (ACSL, PAS_chr4_0352 and PAS_chr2-1_0785), long-chain fatty acid import protein (ABCD, PAS_chr3_0822 and PAS_chr2-2_0272), 2, 4-dienoyl-CoA reductase (PDCR, PAS_chr3_0975), acyl-CoA oxidase (ACOX, PAS_chr-4_0538) and acetyl-CoA acyltransferase 1 (ACAA1, PAS_chr2-2_0267) are all involved in β-oxidation (including β-oxidation of unsaturated fatty acids) and were all significantly upregulated during the GM phase (*p*-Value < 0.05 and log2 fold changes > 1.00) (Figure 4 and Supplementray Table S1). The upregulation of ACSL and ABCD proteins suggests that long-chain fatty acids most likely serve as the predominant source of acetyl-CoA.

The acetyl-CoA generated from β-oxidation can be exported from peroxisomes by two different methods. Firstly, acetyl-CoA is transported after conjugation with carnitine by carnitine acetyltransferase, Cat2, to mitochondria, where it binds to carrier protein Crc1. The second possible destination of acetyl CoA is the glyoxylate cycle, where SUS is the final product (Van Roermund et al., 2003). Because peroxisomes do not penetrate to dinucleotides (i.e., FAD) and acetyl-CoA (Hiltunen et al., 2003), several shuttles are required to maintain the cofactor pool. In this work, FAD-containing enzymes [i.e., acyl-CoA oxidase (ACOX) and alcohol oxidase (AOX1 and AOX2)] were assembled with associated cofactors in the cytosol, and subsequently reoxidized into the peroxisome; the resultant products were combined with the ROS metabolism protein, PXMP4, generating H_2_O_2_ (Cámara et al., 2017).

The upregulation of the adenine nucleotide transporter PMP34 is also essential for the transfer of acetyl-CoA into the glyoxylate cycle. Therefore, most of the acetyl-CoA generated from long-chain fatty acids in this study is likely to have entered the glyoxylate cycle (Rußmayer et al., 2015). Furthermore, the upregulation of ACAA1, a major contributor to the synthesis of triacylglycerols, is instrumental in lipid metabolism (i.e., sphingolipids, phospholipids). Moreover, upregulation of PMXP4 and its binding partner PEX19, which may replace PMP20, can assist ACAA1 in the removal of oxidized lipids to maintain the peroxisomal membrane integrity (Aksam et al., 2008). Stringent regulation of fatty acid metabolism may occur due to the limited availability of acetyl-CoA. This may occur due to the increased anabolic demand for acetyl-CoA as a building block (e.g., for membrane lipid synthesis needed to increase secretory capacity) and fuel for the TCA cycle, i.e., for energy production (Klein et al., 2014). Another possibility is that β-oxidation involved during both pexophagy and mitophagy facilitates the remodeling of organelles as well as the degradation of damaged peroxisome membranes after methanol induction (Vanz et al., 2012; Rußmayer et al., 2015). However, compared with the GM phase, ACSL, ABCD, PDCR, ACOX, ACAA1 and PMXP4 are all down-regulated throughout the methanol induction phase, especially after approximately 72 h of methanol induction. This latter result is almost consistent with data published by Liang et al. (2012) and Rußmayer et al. (2015). It has previously been shown that the use of methanol enables *P. pastoris* cells to grow normally using energy provided by the oxidation of alcohol. However, this latter energy provision strategy does not appear to result in the production of excess carbon and the subsequent assimilation of this carbon into storage reservoirs. As a result, non-polar lipid synthases are down-regulated (Prielhofer et al., 2015; Rußmayer et al., 2015).

These proteins are significantly upregulated during the adaptation phase following the addition of the GM phase, where more acetyl-CoA is produced by beta-oxidation than during the glycerol feed stage. Although our fermentation conditions differed from those used in studies that analyzed metabolic flux of recombinant protein-secreting *P. pastoris* grown on glucose:methanol mixtures (Jordà et al., 2012, 2013), it can be speculated that the metabolic flux of acetyl-CoA in our study increases from the glycerol feed stage to the GM stage. Therefore, we speculate that by increasing the acclimatization period associated with the addition of the GM phase in our high-cell-density fed-batch culture, peroxidases are given the time that is necessary to resist methanol toxicity and additional adverse effects, while also being afforded the opportunity to facilitate self-repair mechanisms. Thus, combined with the analysis of the 100-L fermentation with/without GM phase (Supplementary Figure S1), we believe that this adaptation period promotes the production of exogenous recombinant proteins during the methanol induction phase.

### The Upregulated Genes of the MAPK Signal Pathway May Be Involved in Cell Growth and *AOX1* Promoter Regulation

**FIGURE 5:**
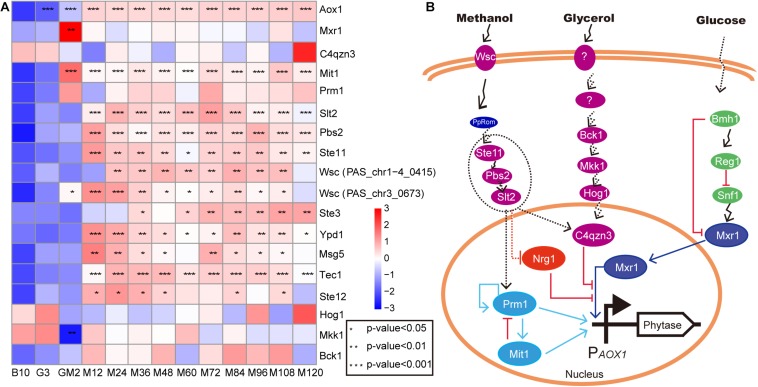
The upregulated genes of MAPK signal pathway involve in cell growth. **(A)** heat map of related genes involved in cell growth and *AOX1* promoter regulation. **(B)** the predicted regulatory relationships for the *AOX1* promoter. The proteins in the dashed ellipse represent proteins that are due to be knocked out and overexpressed in subsequent verification experiments. The methanol regulatory relationship predictions were generated during this study, while the predictions for glycerol (Shen et al., 2016) and glucose (Parua et al., 2012) were reported by other researchers. Purple represents the protein in the MAPK pathway, the dotted line indicates the possible relationship, and the solid line indicates the experimentally validated relationship.

Yeast cellular stress responses that are elicited as a consequence of adverse environmental conditions are mediated by specific signaling pathways; most of these pathways are associated with MAPK cascades. The activation of MAPK induces changes in the global transcriptome, generating efficient adaptive responses that guarantee cell survival (Sanz et al., 2016). In the current study, the addition of methanol, which adversely affects yeast cell growth, resulted in significant upregulation of MAPK signaling pathway-related gene expression during methanol induction (Figure 2). KEGG data reveal that nine upregulated proteins were annotated into the MAPK pathway of *P. pastoris* during the induction period following methanol addition (Figure 5A and Supplementary Table S2). In *Saccharomyces cerevisiae*, stressful conditions that result in damage to the cell wall, an essential structure for cellular viability, trigger the cell wall integrity pathway (CWI) coordinated by the MAPK Slt2 (Levin, 2011). In this study, Slt2 was significantly upregulated during the entirety of the methanol induction phase (*p*-Value < 0.001 and log2 fold changes > 1.48) (Figure 5A and Supplementary Table S2); the Wsc family sensors (Ohsawa et al., 2017) were also significantly upregulated in this regard (*p*-Value < 0.05 and log2 fold changes > 0.75) (Figure 5A and Supplementary Table S2). Although Hog1, a central regulator of osmotic adaptation in yeast, was not significantly upregulated in this study, the Ste11-Pbs2-Hog1 MAPK and Ssk2/Ssk22-Pbs2-Hog1 MAPK cascades (Tatebayashi et al., 2020) have been reported to be activated by osmotic sensors when cells are exposed to high osmotic pressure, suggesting that both Ste11 and Pbs2 are involved in the osmotic stress response (Zarrinpar et al., 2004). The MAPK that replaced Hog1 upregulation in this study was Slt2. Following annotation of the KEGG database, it was observed that Slt2 was enriched in the autophagy and mitophagy pathways (Supplementary Table S2). Meanwhile, Wsc proteins, Ste11, Pbs2, and Slt2 were all enriched in the mitophagy pathway in this study (Supplementary Table S2), suggesting that the Ste11-Pbs2-Slt1 MAPK cascade may be involved in the mitophagy pathway in response to adverse reactions during the pure methanol growth stage. Additionally, unlike *Saccharomyces cerevisiae*, the upregulated MAPK module in *P. pastoris* includes the MAPKKK Ste11, the MAPKK Pbs2, and the MAPK Slt2; these proteins stimulate the synthesis of transcription factors responsible for most of the transcriptional output of the CWI pathway following cell wall stress. Thus, similar to results reported by Shen et al. (2016), Hog1, Mkk1, 2, Pbs2, and Bck1 in the MAPK signaling pathway, are involved in *P. pastoris* cellular growth. The upregulated MAPK signaling pathway may be involved in maintaining cellular growth to resist the damage caused by methanol.

Since most foreign genes expressed in *P. pastoris* are transcribed under the control of the promoter from the *P. pastoris AOX1* gene, transcription in response to methanol is a key feature of this expression system (Lin-Cereghino et al., 2006). In this study, we investigated the expression of the genes, which regulate promoter *AOX1*, including *Mxr1* (Lin-Cereghino et al., 2006; Parua et al., 2012), *C4qzn3* (Parua et al., 2012), *Prm1* (*PpTRM1*) (Sahu et al., 2014; Prielhofer et al., 2015; Wang et al., 2016), and *Mit1* (Wang et al., 2016; Figure 5 and Supplementary Table S2). *Mxr1* (methanol expression regulator 1), whose product, Mxr1, plays an important role in inducing the transcription of *AOX1* and other genes involved in the MUT pathway and *PEX* genes in *P. pastoris*. C4qzn3 protein in *P. pastoris* interacts with Mxr1 through its 14-3-3 binding region via phosphorylation of Ser215 in a carbon source-dependent manner (Parua et al., 2012). Interestingly, C4qzn3 was annotated into the MAPK signaling pathway in this study (Supplementary Table S2). This result suggests that the MAPK signaling pathway is very likely to be involved in the regulation of the *AOX* promoter. However, Parua et al. did not observe C4qzn3-mediated inhibition of Mxr1-dependent gene expression for those genes involved in methanol assimilation pathways (AOX1 and DHAS) and peroxisome biogenesis (PEX8 and PEX14) in response to glycerol in *P. pastoris* (Parua et al., 2012). Nonetheless, the expression of C4qzn3 and Mxr1 exhibited an inversely proportional relationship during the entire fed-batch culture (Figure 5). This indicates that not only does C4qzn3 negatively affect Mxr1 activity, but due to the observation of continuously high yields of phytase, other positive regulatory factors for the *AOX1* promoter might be present. In addition to the inhibition of expression of Mxr1-dependent genes by C4qzn3 under ethanol growth conditions (Parua et al., 2012), it is possible that if C4qzn3 fails to bind to Mxr1, ethanol, upon acting as the carbon source, activated Prm1 and Mit1 to positively regulate the *AOX1* promoter by phosphorylation. An *in vivo* assay showed that the binding of Mit1 and Prm1 to the *AOX1* promoter was carbon-dependent, and the binding avidity was weaker in glycerol, and stronger in methanol (Wang et al., 2016). The methanol induction signal transmitted from Prm1 to Mit1 resulted in the synthesis of large amounts of Mit1 (*p*-Value < 0.001 and log2 fold changes > 3.20), which subsequently induced increased expression of the *AOX1* promoter (Figure 5A and Supplementary Table S2). Almost all protein-protein or protein-DNA interactions that induce expression of the *AOX1* promoter are performed by phosphorylation and dephosphorylation of Ser or Thr; similar occurrences have been observed following binding of C4qzn3 to Mxr1 (Parua et al., 2012) as well as Prm1 to Mit1 (Wang et al., 2016). In this study, the expression of Mxr1 was significantly upregulated during GM (*p*-Value < 0.01 and log2 fold changes ≈ 5.40), and the expression was subsequently maintained at a normal level thereafter. However, Mxr1 levels were still inversely proportional to C4qzn3 levels during the methanol induction period to facilitate the control of Mxr1 expression. Furthermore, it has been reported that methanol stimulation can eliminate the repression of Nrg1 for Mxr1 (Chang et al., 2018). This may be the reason for the normal expression of Mxr1 during the methanol induction stage. Moreover, Mit1 was significantly upregulated during the GM and methanol induction period (*p*-Value < 0.001 and log2 fold changes > 3.20) (Figure 5A and Supplementary Table S2). As such, in our fed-batch fermentation, Prm1 and Mit1 are mainly responsible for regulation of the *AOX1* promoter. However, how methanol activates Prm1 and other transcription factors remains unclear (Wang et al., 2016).

According to previous studies, Wsc family proteins act as methanol sensors during growth on methanol (Sanz et al., 2016). Among these proteins, PpWsc1 and PpWsc3 are involved in the expression of multiple methanol-induced genes (Ohsawa et al., 2017). The Wsc family proteins have also been shown to be involved in sensing methanol and transmitting signals to the nucleus through PpRom2 (Ohsawa et al., 2017). In relation to this current study, it remains to be seen whether the upregulated MAPK pathway participates in signal transmission to the nucleus (Figure 5B). Indeed, verification experiments investigating the knockout and overexpression of Ste11, Pbs2, and Slt2 are still in progress.

### UPR and ERAD Pathways Are Not Significantly Upregulated

ER quality control can be affected by a variety of physiological and pathological conditions. These phenomena can cause the accumulation of misfolded proteins in the ER. “ER stress” refers to an increase in unfolded protein levels; this occurrence can often have deleterious effects on cells. The UPR pathway in eukaryotic cells has evolved to cope with ER stress and facilitate the maintenance of protein homeostasis. Transcriptional upregulation of ER mechanisms involved in folding, lipid biosynthesis, and ERAD machinery is required to coordinate the increase in the ER-folding capacity (Travers et al., 2000) with a concomitant reduction in the folding load through selective mRNA degradation and translational repression (Hollien and Weissman, 2006; Hollien et al., 2009).

**FIGURE 6:**
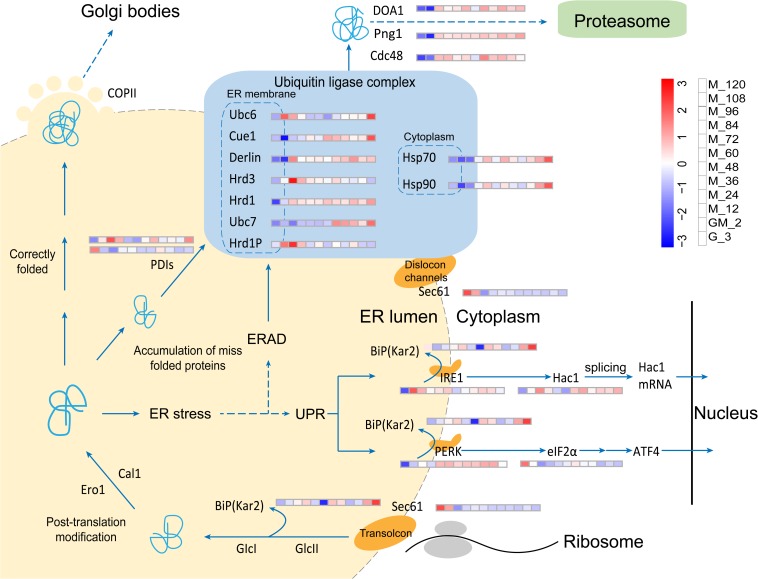
Changes in proteins involved in the UPR and ERAD pathways.

In our fed-batch culture, the *HAC1* gene is always significantly upregulated from glycerol growth to methanol induction (*p*-Value < 0.05 and log2 fold changes > 0.80) (Figure 6 and Supplementary Table S3), indicating that the *HAC1* gene is constitutively expressed. However, the transmembrane ER-stress sensor Ire1 is significantly upregulated during GM and the early stages of methanol induction (M_12) (*p*-Value < 0.05 and log2 fold changes > 0.80) (Figure 6 and Supplementary Table S3). In the cytosol, the unconventional splicing of *HAC1* mRNA is mediated by Ire1. Hac1 translated from the spliced mRNA, the key transcription activator of UPR, targets genes that reduce ER-stress (Van Anken et al., 2014). Likewise, Kar2 (BiP), which can activate Ire1 (Schroder et al., 2003), was not upregulated in this study. In accordance with the results of a study performed by Li et al. (2019), protein disulfide isomerase (PDI, PAS_chr4_0844), which catalyzes appropriate disulfide bond formation during protein folding, is only significantly upregulated (Mares and Ramos, 2018) prior to and at the end of methanol induction (*p*-Value < 0.05 and log2 fold changes > 0.75) (Figure 6 and Supplementary Table S3), while another PDI (PAS_chr1−3_0125) is significantly down-regulated during GM and the methanol induction stage (*p*-Value < 0.05 and log2 fold changes < −0.60) (Figure 6 and Supplementary Table S3). These results indicate that the accumulation of misfolded proteins (phytase) in the ER is not serious, maybe due to the addition of the GM stage in our fed-batch cultivation. Furthermore, Sec61is the major constituent of the eukaryotic ER protein-translocation channel; ER transmembrane kinase/endonuclease IRE1 detects unfolded proteins in the ER. Wheeler and Gekakis carried out a homologous mutation experiment on yeast Sec61p (Y345H) and found that this mutation increased the sensitivity of the yeast strain to ER stress factors while also reducing the activity of IRE1 (Wheeler and Gekakis, 2012). Furthermore, they found that the Sec61p mutation resulted in a delay to the degradation of the model ERAD substrate. In this study, Sec61 is significantly down-regulated during the period of methanol induction (*p*-Value < 0.01 and log2 fold changes < −1.10) (Figure 6 and Supplementary Table S3). As a consequence, Ire1 might not be appropriately activated and the degradation of misfolded substrates in ERAD may be delayed (Wheeler and Gekakis, 2012).

PERK is a type I transmembrane protein commonly found in metabolites. This latter protein is partially similar to Ire1, but is not upregulated in *P. pastoris* (Gardner et al., 2013). PERK also phosphorylates eIF2α, a eukaryotic translation initiation factor. Phosphorylation of eIF2α results in reduced general protein synthesis, which reduces the protein load into the ER (Gardner et al., 2013). However, eIF2α is significantly down-regulated starting from GM (*p*-Value < 0.01 and log2 fold changes < −1.00) (Figure 6 and Supplementary Table S3), suggesting that the levels of the substrate phosphorylated by PERK are reduced. This latter reduction results in a decline in the levels of the phosphorylated form of elFα, a phenomenon that causes a reduction in general protein synthesis inhibition. Although Hrd1p and Png1 were upregulated during the methanol induction phase in this study, the expression of other components including Hsp70, Hsp90, Ubc6, Ubc7, and Hrd3 in the E3 Ubiquitin Ligase complex (a central organizer of the ERAD pathway) (Smith et al., 2011) is not consistent with the former observation (Figure 6 and Supplementary Table S3). Thus, the UPR and ERAD pathways were not significantly regulated in this study. The UPR and ERAD pathways are dynamic responses required for the coordinated disposal of misfolded proteins even in the absence of acute stress (Travers et al., 2000). However, the majority of the proteins involved in the UPR and ERAD pathways are upregulated at the M_120 (Figure 6 and Supplementary Table S3), and C4qzn3 which inhibits the *AOX*1 promoter (Parua et al., 2012), is significantly upregulated, indicating that the phytase yield in *P. pastoris* may become limited, leading to the accumulation of unfolded proteins.

### The Upregulation of the Autophagy Pathway

**FIGURE 7:**
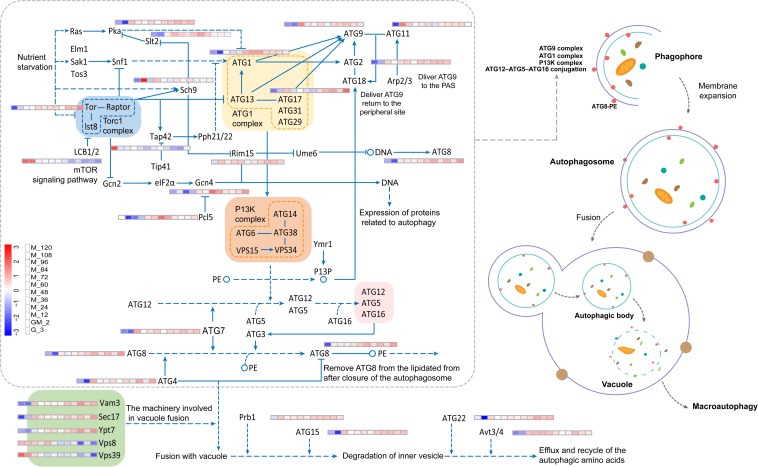
Changes in proteins involved in the autophagy pathway.

Autophagy is a highly conserved catabolic pathway that maintains cellular homeostasis by removing damaged organelles and misfolded proteins, and replenishing biosynthetic precursors during starvation (Davies et al., 2015). In yeast, the limited number of genes that were shown to have products primarily involved in autophagy (and also in selective microautophagy) were unified under the name autophagy-related (*ATG*) (Wen and Klionsky, 2016). Starvation induces autophagy in part through deactivation of the nutrient-sensing TORC1 kinase. Deactivation of TORC1 promotes the recruitment of the most upstream set of autophagy-regulating proteins, members of the ATG1 complex, to the phagophore assembly site (PAS) (Hu et al., 2019). ATG1 not only promotes autophagy induction, but may also facilitate late stages of auto-phagosome biogenesis (Kijanska and Peter, 2013). In this study, the autophagy-related pathways like autophagy-yeast, mitophagy-yeast, and autophagy-other were almost all significantly upregulated during the methanol induction stage (*p*-Value < 0.05 and log2 fold changes > 0.50) (Figure 2), and ATG1 complex proteins and other autophagy-related proteins were significantly upregulated (*p*-Valuer < 0.05 and log2 fold changes > 0.90) apart from Tap42, Vps8, LCB1/2, and Vps39 (Figure 7 and Supplementary Table S4). A report by Yorimitsu et al. (2009) revealed a previously unknown negative role for the Tap42-phosphatase pathway in the regulation of autophagy. Rab7-binding HOPS promotes endolysosomal trafficking in all eukaryotes. However, Vps8 overexpression abolishes the late endosomal localization of HOPS-specific Vps41/Lt and prevents HOPS assembly (Lorincz et al., 2019). Thus, Vps8 negatively regulates HOPS by outcompeting Vps41 to inhibit the autophagy pathway, leading to inhibition of the autophagy pathway. A separate study revealed that a direct interaction between LGG-2 and the HOPS complex subunit Vps39 is involved in autophagosome degradation (Manil-Segalen et al., 2014). It is also known that proteins Tap42, Vps8, LCB1/2, and Vps39 negatively regulate the autophagy pathway. Therefore, the down-regulation of these proteins observed in this study is likely to be beneficial to autophagy-related pathways (Figure 7 and Supplementary Table S4). Observations under the electron microscope revealed that invagination of vacuoles during peroxisome degradation by microautophagy (micropexophagy) was a normal occurrence (Vanz et al., 2012; Burnett et al., 2015; Tripathi et al., 2016). Micropexophagy requires high levels of ATP (Ano et al., 2005) and appropriate levels are most likely available in the methanol induction phase primarily following dissimilatory methanol catabolism. They predominantly observed vacuolar enclosure for peroxisomes but not in HBsAg deposits (Vanz et al., 2012), indicating that vacuolar degradation pathways are not induced by recombinant protein accumulation; this phenomenon may have been facilitated by damaged peroxisomes. Autophagy of peroxisomes has been reported during the conversion of methanol to ethanol or glucose in *P. pastoris* (Vanz et al., 2012); however, autophagy was not reported when the same cells were grown in methanol alone. These events suggest that the constitutive autophagic cycle of peroxisomes may represent a housekeeping strategy employed by *P. pastoris* during methanol growth. This recycling strategy helps cells to deal with damage caused by reactive oxygen species produced by the oxidation of methanol; this is consistent with research published by Vanz et al. (2012). Indeed, constitutive pexophagy has been shown to be vital for the methylotrophic yeast species *Hansenula polymorpha* during growth on methanol. The latter experiment demonstrated that mutant cells with defects in autophagy displayed reduced vitality (Aksam et al., 2007) and autophagic degradation are rapidly performed in damaged peroxisomes in *H. polymorpha* (Van Zutphen et al., 2011). Thus, the autophagy pathways (Figure 2) and the ATG1 series proteins (Figure 7 and Supplementary Table S4) are almost all significantly upregulated during the ongoing methanol induction phase; however, the phytase yield always increased during the same phase. These results indicate that the upregulation of the autophagy pathway may not be related to phytase production but to the degradation of damaged peroxisomes.

## Conclusion

*Pichia pastoris* is a well-established host system for heterologous protein expression in industry. Because this system still has many shortcomings, we conducted a 10000-L high-cell-density fed-batch fermentation and compared the results with previous studies (Liang et al., 2012; Vanz et al., 2012; Hesketh et al., 2013). Importantly, we increased the GM phase before the methanol induction stage. We subsequently investigated changes in the metabolic pathways of *P. pastoris* in all stages of our fed-batch culture by RNA-seq technology. The metabolic pathway changes during the GM phase predominantly occurred between the transitions to the glycerol growth phase and the methanol induction phase (Figure 2). In relation to carbon metabolism pathways, glycerol kinase (GUT1) and alcohol dehydrogenase (ADH) were remarkably upregulated during the adaptation period of GM, and AOX1 was also significantly upregulated during this stage (*p*-Value < 0.001 and log2 fold changes ≈ 6.30) (Figure 3). Furthermore, proteins associated with the fatty acid metabolism pathway are significantly upregulated during the adaptation phase of GM. These results indicate that the GM phase promotes the growth of *P. pastoris* cells (Cos et al., 2006; Potvin et al., 2012) when recombinant protein synthesis is initiated (Figure 1). The upregulated MAPK module in the MAPK signaling pathway includes the MAPKKK Ste11, the MAPKK Pbs2, and the MAPK Slt2. These observations suggest that the MAPK signaling pathway may be involved in maintaining cell growth by resisting damage caused by methanol (Shen et al., 2016). In addition, we predict that the MAPK signaling pathway might be primarily responsible for the methanol-stimulated signaling of regulated factors of the *AOX1* promoter (Figure 5B). Moreover, following analysis of the UPR and ERAD pathways as well as the autophagy pathway, we observed that phytase enzyme activity was still elevated in the later stages of fermentation (Figure 1); thus, the upregulation of the autophagy pathway during the methanol induction phase may be related to the degradation of damaged peroxisomes but not to the production of phytase. Our results describe the metabolic characteristics of *P. pastoris* during heterologous protein production under high-cell-density fed-batch cultivation. The study also provides a significant platform to further analyze *P. pastoris* protein expression, regulation and secretory mechanisms.

## Data Availability Statement

The gene expression data generated during this study have been submitted to the Gene Expression Omnibus (GEO) database of NCBI; a GEO accession number GSE142326 was assigned to these data.

## Author Contributions

CZ and ZH designed the manuscript. CZ performed the data analysis and wrote the manuscript. YM completed the Figures 6, 7. HM collated the samples. XT and BX performed the fermentation and tested the biomass and enzyme activity. QW and YLM prepared the experimental materials. ZH and CZ revised the manuscript. All authors read and approved the final manuscript.

## Conflict of Interest

The authors declare that the research was conducted in the absence of any commercial or financial relationships that could be construed as a potential conflict of interest.

## Footnotes

1 https://www.ncbi.nlm.nih.gov/genome/?term=GS115

2 https://www.ncbi.nlm.nih.gov/geo/query/acc.cgi?acc=GSE142326

3 http://www.genome.jp/kegg/

## Abbreviations

6PG: gluconate-6P
CTS: citrate synthase
DAK: triose/dihydroxyacetone kinase
DAS: dihydroxyacetone synthase
DHA: dihydroxyacetone
DHAP: dihydroxyacetone-P
DLD: dihydrolipoamide dehydrogenase
E4P: erytgrose-4P
ENOI: enolase I
F1,6BP, fructose-1: 6-P2
F6P: fructose-6P
FBAII, fructose-bisphosphate aldolase: class II
FBPI: fructose-1,6-bisphosphatase I
FCH: S-formylglutathione hydrolase
FDH: formate dehydrogenase
FLD1: S-(hydroxymethyl)glutathione dehydrogenase
Form: formaldehyde
FUM: fumarate
G1,3P: glycerate-1,3P2
G2P: glycerate-2P
G3P: glycerate-3P
G6P: glucose-6P
GAP: glyceraldehyde-3P
GAPD: glyceraldehyde 3-phosphate dehydrogenase
GPI: glucose-6-phosphate isomerase
GS-CH_2_OH: S-hydroxymethylglutathione
GS-CHO: S-formylglutathione
ISO: isocitrate
MAL: malate
MDH: malate dehydrogenase
ODE: 2-oxoglutarate dehydrogenase E2
OXA: oxaloacetate
OXAL: oxalosuccinate
PDE: pyruvate dehydrogenase E
PEP: phosphoenolpyrvate
PEX: peroxisomal
PGK: phosphoglycerate kinase
R5P: ribose-5P
RKI: ribose-5-phosphate ketol-isomerase
Rul5P: ribulose-5P
S7P: sedoheptulose-7p
SCS1: succinyl-CoA synthetase 1
SCS2: succinyl-CoA synthetase 2
SDH: succinate dehydrogenase
SUC: succinate
TAL: transaldolase
TK: transketolase
X5P: xylulose-5P.
