# Supplementary material for: Transcriptomic Analysis of Pichia pastoris (Komagataella phaffii) GS115 During Heterologous Protein Production Using a High-Cell-Density Fed-Batch Cultivation Strategy
Source: Front Microbiol. 2020 Mar 20;11:463. doi: 10.3389/fmicb.2020.00463 (PMC7098997; doi:10.3389/fmicb.2020.00463)

Supplementary information

Table S1. The related proteins involved in carbon metabolism and oxidative stress metabolic pathway.

Table S2. The related genes involved in cell growth and AOX1 promoter regulation.

Table S3. The related genes involved in UPR and ERAD pathways.

Table S4. The related genes involved in the autophagy pathway.

Figure legend

Figure S1. Biomass and phytase enzyme activity with/without GM phase in the 100-L fermentation.

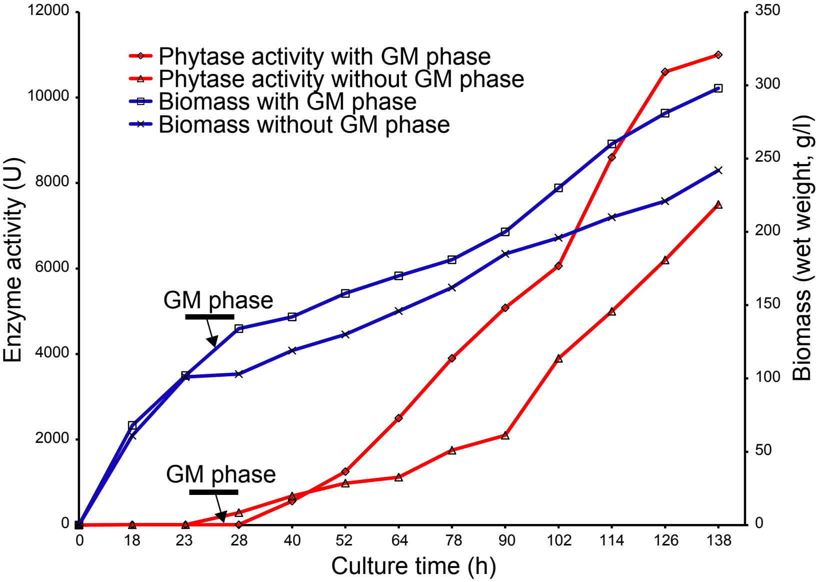

Supplement: Supplementary file 1 [file Data_Sheet_1.PDF]
